# The LPS-Induced Transcriptional Upregulation of the Chicken Lysozyme Locus Involves CTCF Eviction and Noncoding RNA Transcription

**DOI:** 10.1016/j.molcel.2008.07.023

**Published:** 2008-10-10

**Authors:** Pascal Lefevre, James Witham, Claire E. Lacroix, Peter N. Cockerill, Constanze Bonifer

**Affiliations:** 1 Section of Experimental Haematology, Leeds Institute of Molecular Medicine, University of Leeds, Wellcome Trust Brenner Building, St. James's University Hospital, Leeds LS9 7TF, UK

**Keywords:** RNA, DNA, MOLIMMUNO

## Abstract

Transcription of the lysozyme gene is rapidly induced by proinflammatory stimuli such as treatment with bacterial lipopolysaccharide (LPS). Here we show that this induction involves both the relief of repression mediated by the enhancer-blocking protein CTCF that binds to a negative regulatory element at −2.4 kb, and the activation of two flanking enhancer elements. The downstream enhancer has promoter activity, and LPS stimulation initiates the transient synthesis of a noncoding RNA (LINoCR) transcribed through the −2.4 kb element. Expression of LINoCR is correlated with IKKα recruitment, histone H3 phosphoacetylation in the transcribed region, the repositioning of a nucleosome over the CTCF binding site, and, eventually, CTCF eviction. Each of these events requires transcription elongation. Our data reveal a transcription-dependent mechanism of chromatin remodeling that switches a *cis*-regulatory region from a repressive to an active conformation.

## Introduction

The inflammatory response requires the rapid activation of proinflammatory genes in cells of the innate immune system. Toll-like receptors play a critical role in responding to microbial components such as lipopolysaccharide (LPS) by activating common signal transduction pathways such as the mitogen-activated protein kinase family (Guha and Mackman, 2001; Ronni et al., 2003). Specific transcription factors, such as NF-κB/Rel, AP-1 (Jun/Fos), and CAAT enhancer binding protein (C/EBP) families are the downstream targets of LPS-induced signaling (Guha and Mackman, 2001; Plevy et al., 1997; Stein and Baldwin, 1993).

**Figure 1:**
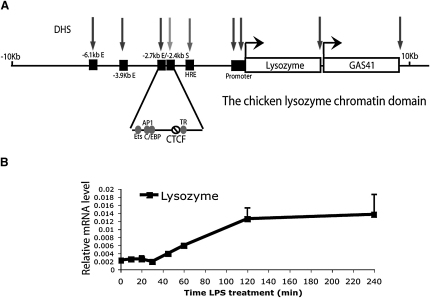
The Lysozyme Gene Is LPS Inducible (A) General organization of the lysozyme locus. (B) Time course of lysozyme mRNA expression in HD11 cells following LPS stimulation. Results are expressed relative to GAPDH expression. Error bars represent ± SD from three independent experiments.

The chicken lysozyme gene is a well-studied model to investigate the effects of proinflammatory stimuli on gene expression. It is upregulated during macrophage differentiation and reaches its highest expression level in LPS-stimulated macrophages. Transcription is controlled by three enhancers located 6.1, 3.9, and 2.7 kb upstream of the transcription start site, a complex promoter, and a negative regulatory element at −2.4 kb (Bonifer et al., 1997). The −2.4 kb element was shown to have silencer activity via its ability to block the activity of the −2.7 kb enhancer and lysozyme promoter activity independently of its position and orientation (Baniahmad et al., 1987, 1990). Transcription factor recruitment occurs in several steps, with the early acting transcription factors such as NF1 and Fli-1 binding first to the −6.1 and −3.9 kb enhancers, followed by the recruitment of CREB-binding protein (Kontaraki et al., 2000; Lefevre et al., 2003). LPS stimulation leads to an additional recruitment of C/EBPβ and significant alterations in chromatin structure at the enhancers and promoter (Kontaraki et al., 2000; Lefevre et al., 2005, 2003), such as a switch in the pattern of DNase I hypersensitive sites (DHSs). Prior to LPS induction, DHSs are present at the −2.4 kb element and at the −6.1 and −3.9 kb enhancers. Following stimulation, the DHS at the −2.4 kb element disappears, and two new DHSs appear at the −2.7 kb enhancer and at a hormone response element (HRE) at −1.9 kb. In addition, this region contains specifically positioned nucleosomes, which are remodeled after LPS stimulation (Hecht et al., 1988; Huber et al., 1995, 1996; Kontaraki et al., 2000) (Figure 1A). However, neither the exact kinetics of chromatin modification nor the nature of interdependence between these regulatory elements is known.

CTCF is a highly conserved, ubiquitously expressed zinc finger protein that plays a critical role in transcriptional regulation in vertebrates with diverse functions including promoter repression or activation, enhancer-blocking activity, and chromatin insulation (Bell et al., 1999; Klenova et al., 2002; Ohlsson et al., 2001). Recently, 13,804 CTCF-binding sites within the human genome were identified that are largely invariant across different cell types (Kim et al., 2007), suggesting that CTCF is crucial for genome-wide transcription regulation. The −2.4 kb element was one of the first identified targets of the transcription factor CTCF. It is a composite regulatory element encompassing a thyroid hormone response element (TRE) located next to the CTCF-binding site (Baniahmad et al., 1990), and it is also functional in insulator assays (Lutz et al., 2003). Binding of the thyroid hormone receptor to the TRE abrogates the enhancer-blocking effect of CTCF in insulator assays, although it remains tethered to its binding site. The exact mechanism leading to the abrogation of CTCF-mediated insulator activity after thyroid hormone treatment is not understood, but specific phosphorylation and poly-ADP-ribosylation are two reported CTCF modifications important for changes in CTCF functions (El-Kady and Klenova, 2005; Yu et al., 2004). In this respect, it is interesting to note that CTCF has recently been shown to recruit the cohesin complex to genomic sites and that this recruitment is crucial for the insulator activity of CTCF (Parelho et al., 2008; Wendt et al., 2008).

In this report, we studied the mechanism of lysozyme gene activation by proinflammatory stimuli. We show that CTCF recruits cohesin to the lysozyme element and that LPS stimulation results in the eviction of the CTCF/cohesin complex. This leads to the abrogation of CTCF-mediated repression, and we demonstrate that this eviction relies on transcription-dependent nucleosome remodeling.

## Results

### Induction of Lysozyme mRNA Expression Is Preceded by Recruitment of Transcription Factors and RNA Polymerase II to Upstream Regulatory Elements

Our previous work demonstrated that LPS treatment causes extensive chromatin modifications within the 5′ region of the lysozyme locus (Huber et al., 1995, 1996; Lefevre et al., 2005, 2003). To investigate the molecular mechanism of this process, we studied the order of events occurring within chromatin leading to the upregulation of the gene after LPS stimulation. As a model, we employed the HD11 chicken monocyte cell line, which upon LPS treatment undergoes terminal differentiation and growth arrest. The genomic and chromatin organization of the lysozyme locus in macrophages is summarized in Figure 1A (Chong et al., 2002b; Huber et al., 1995). We first examined the timing of upregulation of lysozyme mRNA levels following LPS stimulation (Figure 1B). After stimulation, lysozyme mRNA levels remained unchanged for at least 30 min but then doubled after a further 15 min and after 2 hr of LPS treatment reached a plateau corresponding to five to ten times the level present in the untreated control.

**Figure 2:**
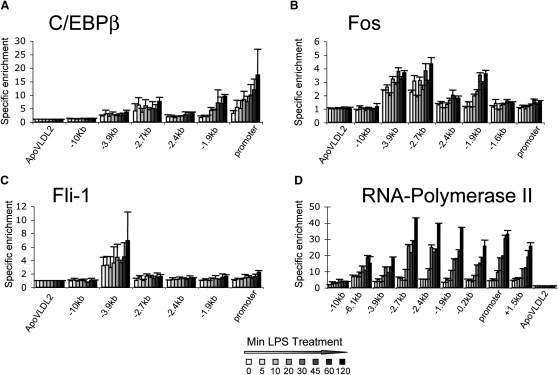
ChIP Assays of Time Course of Transcription Factor Binding and RNA Pol II Recruitment Following LPS Stimulation Chromatin from LPS-treated HD11 cells was precipitated with antibodies for (A) C/EBPβ, (B) Fos, (C) Fli-1, or (D) RNA Pol II. Increasing incubation times in minutes are illustrated by increasing gray intensity from uninduced cells (no LPS) in white to 2 hr LPS induction in black (120 min). Data are analyzed by real-time PCR with primers named according to their distance from the transcription start site. The amount of PCR product for each primer is expressed relative to a genomic DNA serial dilution. Data are expressed using the following formula (specific IP/IgG)_x_/(specific IP/IgG)_apovldl2_ (with X representing a specific primer and apovldlII the promoter of an hepatocyte-specific gene, used as negative control), and error bars represent ± SD from three independent experiments.

We next measured transcription factor and RNA polymerase II (RNA Pol II) recruitment within the 5′-regulatory region during a time course of induction by chromatin immunoprecipitation (ChIP) in HD11 cells. LPS stimulation induces a redistribution of C/EBPβ protein from the cytoplasm to the nucleus (Katz et al., 1993). We therefore expected a rapid increase in C/EBPβ binding to lysozyme *cis*-regulatory elements. This was found at the promoter, where C/EBPβ occupancy increased after 20 min of induction (Figure 2A). However, C/EBPβ occupancy changed only weakly at the −2.7 kb enhancer and only after 2 hr (Figure 2A). In contrast, C/EBPβ progressively accumulated at the −1.9 kb HRE with similar kinetics as observed at the promoter (Figure 2A). We next focused on AP-1 as another LPS inducible transcription factor known to target the lysozyme gene (Grewal et al., 1992; Phi van, 1996). Using an antibody that recognizes all Fos family members, we detected AP-1 binding at the −3.9 and −2.7 kb enhancers in HD11 cells (Figure 2B), which increased after LPS stimulation. In addition, as observed for C/EBPβ, Fos/AP-1 was undetectable at the HRE prior to LPS treatment but was bound 20 min after stimulation, reaching a plateau after 45 min (Figure 2B). Furthermore, Fos recruitment at the −2.7 kb enhancer was faster than C/EBPβ. As expected (Lefevre et al., 2003), the Ets family member Fli-1 only bound to the −3.9 kb element, and this binding was not LPS inducible (Figure 2C).

The level of RNA Pol II recruitment to the lysozyme gene paralleled the accumulation of C/EBPβ and Fos/AP-1 at the −1.9 kb element and the promoter. These data are consistent with mainly transcriptional activation of lysozyme gene expression after LPS stimulation. After 20–30 min following LPS treatment, RNA Pol II was recruited to *cis*-regulatory elements located within 3 kb upstream of the transcription start (Figure 2D). These data indicate that (1) transcription factor binding and RNA Pol II recruitment are concomitant and (2) changes observed after brief LPS stimulation are limited to the first 3 kb of the 5′-regulatory region.

### LPS Induces CTCF Eviction and Nucleosome Remodeling within the HRE/−2.4/−2.7 kb Region

**Figure 3:**
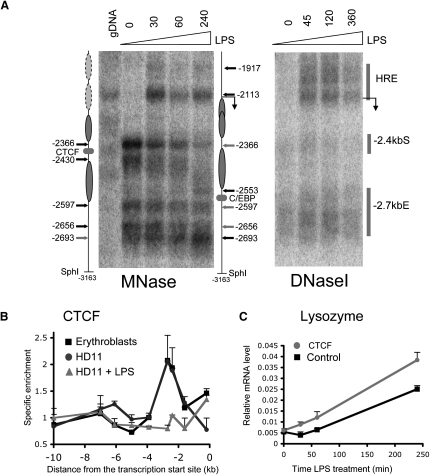
The CTCF-Binding Site Is Progressively Covered by a Nucleosome, and CTCF Is Evicted from Its Binding Site (A) Nuclease digestion analysis of a region 3 kb upstream of the transcription start site. Genomic DNA isolated from DNase I (right panel) and MNase (left panel)-treated chromatin, as well as control genomic DNA digested with MNase, were analyzed as described in the Experimental Procedures. HD11 cells were treated with LPS for 0–240 min. The deduced nucleosomal organization is depicted on the left part of the figure for nontreated HD11 cells and on the right for HD11 cells treated with LPS for 4 hr. The transcription start site deduced from the 5′-RACE experiment is also indicated. (B) CTCF ChIP assays of HD37 cells (dark squares), HD11 cells (gray circles), and HD11 cells treated with LPS for 24 hr (gray triangles). Error bars represent ± SD from three measurements. Data are representatives of three independent experiments. (C) Time course of LPS-induced lysozyme gene expression 24 hr after CTCF knockdown (gray circles) compared to the control (black squares). Data are expressed relative to GAPDH. Error bars represent ± SD from three independent experiments. For all other explanations, see the legend of Figure 2.

We next focused on chromatin changes within the 3 kb upstream region during a time course of LPS induction. To this end, we performed micrococcal nuclease (MNase) mapping of nucleosome positions as well as DHS analysis (Figure 3A). At the −1.9 kb HRE, we noted the rapid induction of a strong DHS with a parallel increase in hypersensitivity at the −2.7 kb enhancer. This DHS existed as two closely linked regions centered at −1.9 and −2.1 kb that were hypersensitive to both DNase I and MNase. A preferential MNase digestion site detected in the genomic DNA control at −2113 bp and the MNase site downstream were both protected by nucleosomes in nonstimulated cells and became fully accessible after 30 min LPS treatment.

In the −2.4/−2.7 kb region, we noted that in untreated HD11 cells the MNase hypersensitive sites at −2366 and −2430 bp mark the boundary of the CTCF complex occupying a ∼80 bp nucleosomal linker region. The MNase sites at −2553 and −2693 bp flank a DHS indicative of a protein complex interacting with the −2.7 kb enhancer (Figure 3A). These results are consistent with the presence of a positioned nucleosome between −2430 and −2597 bp. After LPS induction, several chromatin features in this region were altered. Of the two strong MNase hypersensitive regions flanking the CTCF binding site, only one remained, albeit weakly, at −2366 bp and an additional region at −2553 bp downstream of the C/EBP binding site at the −2.7 kb enhancer was preferentially cleaved. These changes indicated the displacement of the positioned nucleosome located at −2430 to −2597 bp to position between −2366 to −2553 bp, now occupying the CTCF binding site. This was confirmed by densitometric analysis of the MNase data after correction for the MNase sensitivity of genomic DNA control digests (Bert et al., 2007) (see Figure S1 available online). This result is important because CTCF cannot bind to nucleosomal DNA (Kanduri et al., 2002), and this movement also frees up the C/EBP binding site located at −2562 bp. Interestingly, in contrast to the −1.9 kb region, nucleosome reorganization in the −2.4/−2.7 kb region required 1–4 hr of LPS stimulation.

To directly test the consequences of this nucleosome movement for CTCF binding, we measured CTCF binding by ChIP assays and real-time PCR after exposure to LPS. As expected, CTCF bound to the −2.4 kb element in untreated HD11 cells and also in the erythroblast cell line HD37. However, after LPS treatment, CTCF binding disappeared (Figure 3B). No change in binding was seen at another CTCF target, the chicken β-globin *cis*-regulatory element HS4 (Bell et al., 1999), confirming that eviction is lysozyme gene specific (Figure S2). Taken together, our data indicate that LPS-induced transcription factor association leads to significant alterations in chromatin architecture that are incompatible with CTCF binding.

We next investigated the consequences of CTCF binding and eviction on lysozyme transcription. We knocked down CTCF using a cocktail of three RNAi molecules, stimulated HD11 cells with LPS 24 hr later, and measured lysozyme mRNA upregulation (Figure 3C). Knockdown of CTCF mRNA and protein (≈60%) was confirmed by real-time PCR and western blotting (Figures S3A and S3B). No difference in lysozyme mRNA levels was measured after 24 hr of knockdown, indicating that CTCF depletion was not sufficient to upregulate lysozyme mRNA expression in the absence of LPS treatment. However, we observed an earlier onset of induction (30 min), indicating a repressive role of CTCF prior to LPS treatment. Taken together, this shows that the upregulation of lysozyme expression is mediated by both CTCF eviction and the LPS-induced recruitment of transcription factors.

### The −1.9 kb DHS Is a Dual Promoter/Enhancer Element in Macrophages, and LPS Stimulation Transiently Increases Transcription from this Element

**Figure 4:**
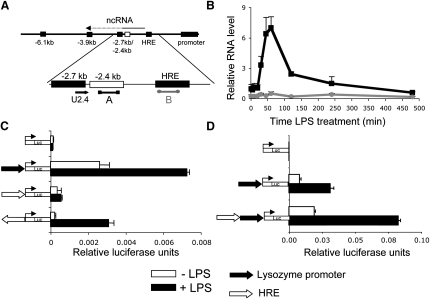
Detection of a LPS Inducible Noncoding RNA Initiating from the −1.9 kb Enhancer/Promoter Element (A) Schematic of the biotinylated primers used to identify LINoCR (depicted as an arrow) and the position of amplicons. (B) Time course of LINoCR expression. HD11 cells were treated with LPS for 0–480 min. cDNA synthesis was performed by using the U2.4 biotinylated primer and a biotinylated primer specific for GAPDH. Real-time PCR was performed using primer A (black) or primer B (gray). Data are expressed relative to GAPDH expression. Error bars represent ± SD from three experiments. (C and D) Transient transfection assays in HD11 cells. The black and white arrows represent the lysozyme promoter and the −1.9 kb element (HRE), respectively. The direction of the arrow illustrates the orientation of the element. Reporter activity was measured with (black bars) or without 7.5 hr of LPS treatment (white bars). Data are expressed relative to a Renilla control, and error bars represent ± SD from three independent experiments.

We previously demonstrated that an increase in histone H3 lysine 4 methylation (H3meK4) correlates with the transcriptional activity of the lysozyme gene (Lefevre et al., 2005). Here we show that within the −1.9 and −2.7 kb regions this increase correlates with RNA Pol II recruitment (Figure 2D). Because many transcripts from eukaryotic genomes correspond to noncoding RNAs (Mattick, 2005), we searched for such transcripts in the lysozyme *cis*-regulatory region by using strand-specific RT-PCR employing biotinylated primers (data not shown). We detected an antisense transcript overlapping the −2.4/−2.7 kb region, but not the −1.9 kb region (Figures 4A and 4B). We named this transcript LINoCR (LPS Inducible NonCoding RNA). Using 5′-RACE and RNase protection assays, we mapped the transcription start site of LINoCR to −2120 bp (Figures S4A and S4B).

LINoCR was detectable after 30 min of LPS stimulation, peaked between 45 min and 1 hr, decreased by 65% after 2 hr, and became undetectable after 8 hr LPS stimulation (Figure 4B). Taking into account that transcription may not be completely shut down, this indicated a half-life of this transcript of 45 min or less. The localization of the transcription start site and the de novo induction of a DHS suggested that the −1.9 kb element was the promoter. We therefore cloned the −1.9 kb element in both orientations upstream of a luciferase gene and assayed reporter gene activity after transient transfection into HD11 cells with and without LPS treatment (Figure 4C). A reporter construct carrying the lysozyme mRNA promoter served as positive control. The −1.9 kb element exhibited promoter activity in the antisense orientation, but only after LPS treatment. The −1.9 kb element has been shown to be a steroid hormone-responsive enhancer element in nonmyeloid cells (Hecht et al., 1988). We placed it upstream or downstream of the lysozyme promoter, and the position-independent activity confirmed that this element is an enhancer in macrophages (Figure 4D and data not shown).

In summary, we conclude that the −1.9 kb element has dual promoter/enhancer function and that LINoCR, transcribed from this element, reads through the −2.4 kb element with a peak of expression correlating with the upregulation of lysozyme gene expression after LPS treatment.

### Histone H3 S10 Phosphorylation Spatially and Temporally Correlates with LINoCR Transcription

**Figure 5:**
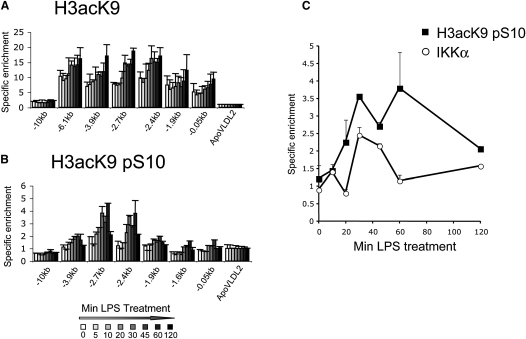
ChIP Assays of the Time Course of Histone H3 K9 Acetylation, S10 Phosphorylation, and IKKα Binding Following LPS Stimulation HD11 cells were treated with LPS, and sonicated chromatin was precipitated with antibodies for histone AcK9H3 (A), dual AcK9 + pS10 ([B] and [C], black square), or IKKα ([C], white circle). For all other details, see legend of Figure 2. (C) Time courses for primer −2.4 Kb. Error bars represent ± SD from three measurements. Data are representative of two experiments.

H3 S10 phosphorylation has been previously observed at inducible genes in response to mitogenic or stress stimuli (Clayton et al., 2000) and is associated with HP1 dissociation from heterochromatin during DNA replication (Hirota et al., 2005; Mateescu et al., 2004). We therefore tested whether this histone modification increased upon LPS induction and whether it correlated with CTCF eviction. To this end, we analyzed changes in both histone H3 K9 acetylation (H3 AcK9) and H3 S10 phosphorylation during LPS stimulation using ChIP assays with antibodies recognizing just H3 AcK9 or both marks together on the same histone tail (Figure 5). H3 AcK9 increased at the −6.1, −3.9, −2.7, and −2.4 kb elements from 30 min following LPS treatment and also slightly at the promoter and the −1.9 kb element (Figure 5A). However, the pattern and timing of dual H3 S10 phosphorylation/K9 acetylation after induction were different. While H3 AcK9 increased throughout the 5′-regulatory region, H3 phosphoacetylation was restricted to the −2.4/−2.7 kb region and appeared only transiently between 30 min and 1 hr (Figure 5B). These data suggest a precise correlation between LINoCR expression and the appearance of the histone H3 S10 phosphorylation mark.

Histone H3 phosphorylation by MSK1/2 has previously been associated with the activation of inducible genes in response to mitogenic or stress stimulation (Clayton et al., 2000), raising the possibility that MSK1/2 was the responsible kinase for histone H3 S10 phosphorylation at the lysozyme locus. However, the MSK1/2 inhibitor H89 did not prevent histone phosphorylation at the −2.4 kb element (data not shown). We found instead that IKKα was transiently recruited to the −2.4 kb element (Figure 5C), as shown for cytokine-induced genes (Yamamoto et al., 2003).

### CTCF Eviction from the −2.4 kb Element Is Transcription Dependent

The experiments described above demonstrated that (1) after LPS induction RNA Pol II was simultaneously recruited to the −1.9 kb region and to the promoter, and (2) the presence of CTCF at the −2.4 kb element delayed LPS-induced lysozyme gene activation. It was also possible that CTCF regulated LINoCR expression. Therefore we knocked down CTCF expression in HD11 cells with and without 1 hr LPS treatment when LINoCR expression was maximal. CTCF knockdown had no effect on LINoCR expression (Figures S3C and S3D), indicating that LINoCR transcription was regulated by the LPS-induced binding of transcription factors to the −1.9 kb element.

**Figure 6:**
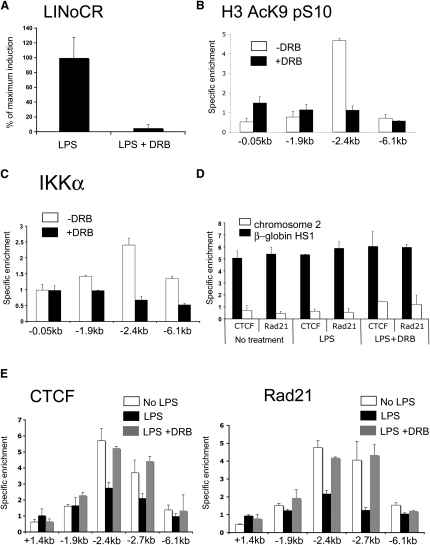
DRB Treatment Blocks IKKα Recruitment, Histone H3 Phosphoacetylation, and CTCF Eviction (A) Real-time PCR assays LINoCR expression in HD11 cells treated with LPS for 1 hr with or without DRB added 15 min after the start of LPS induction. (B) ChIP assay with an anti-histone H3 acK9 + pS10 antibody. HD11 cells were treated with LPS for 1 hr with (black bars) or without (white bars) addition of DRB 15 min after the start of LPS incubation. (C–E) ChIP assay with anti-IKKα antibodies, with anti-CTCF or anti-Rad21 antibodies as indicated. Primary macrophages were incubated without LPS or DRB (white bars) or with LPS for 3 hr with (gray bars) or without (black bars) addition of DRB 15 min after the start of LPS incubation. Data were generated by real-time PCR with primer pairs (B, C, and E) named according to their distance from the transcription start site or (D) corresponding to the CTCF-binding site of the mouse β-globin HS1 (black bars) or a negative control region on chromosome 2 (white bars). For all other explanations, see the legend of Figure 2. Error bars represent ± SD from three measurements. Data are representative of at least two experiments.

The experiments described so far raised the possibility of a direct link between promoter activity at the −1.9 kb element and transcription-associated alterations of histone modification, nucleosome repositioning, and CTCF eviction. The small size of the −2.7/−2.4/−1.9 kb region and the importance of precisely coordinated alterations of chromatin architecture excluded testing this hypothesis by inserting a transcription termination element that in itself spans several hundred base pairs. We therefore blocked transcriptional elongation by using the specific inhibitor 5,6-dichloro-1-β-D-ribofuranosylbenzimidazole (DRB). It has previously been shown that short-term DRB treatment has no influence on RNA-polymerase association and transcription factor binding (Mitchell and Fraser, 2008). The onset of RNA Pol II and transcription factor binding at the −1.9 kb element occurred within 20 min after LPS stimulation (Figure 2). Therefore, HD11 cells were stimulated with LPS for 15 min and then treated with DRB. Under these conditions, transcriptional activation of immediate early genes like cJun and cFos as factors necessary for mediating LPS induction was not affected (Figure S5). In agreement with this idea, nucleosome remodeling and the formation of a DHS at the −1.9 kb element were unaffected (Figure S8). However, DRB treatment completely blocked LINoCR expression, IKKα recruitment, and histone H3 S10/K9 phosphoacetylation at the −2.4 kb element (Figures 6A–6C).

The initial ChIP assays demonstrating CTCF eviction in chicken cells (Figure 2B) were performed using an antibody that is no longer available, so we explored an alternative model system. The complete chicken lysozyme locus is expressed in a position-independent fashion in transgenic mice and adopts the identical chromatin structure as in chicken cells (Bonifer et al., 1990; Huber et al., 1994; Tagoh et al., 2004). We therefore repeated our experiments using primary macrophages from mice carrying a single copy of the chicken lysozyme locus (Chong et al., 2002a). This also enabled us to look for the presence of Rad21, a protein of the cohesin complex (Parelho et al., 2008; Wendt et al., 2008). In primary mouse macrophages, chicken lysozyme mRNA and LINoCR expression followed the same kinetics as in HD11 cells (Figure S6). Both CTCF and Rad21 were detected by real-time PCR at the −2.4 kb element and were both evicted after LPS stimulation (Figure 6C), suggesting that both proteins participate in regulating its activity. However, this did not occur when LPS stimulation was followed by DRB treatment 15 min later (Figure 6C). In contrast, the mouse β-globin *cis*-regulatory element HS1, which is known to bind CTCF (Farrell et al., 2002), also bound Rad21 but did not show any change in the binding of either protein after LPS treatment, again confirming that CTCF and Rad21 eviction were lysozyme specific (Figure 6D). These data suggest that transcription is necessary for the eviction of the CTCF/cohesin complex from its binding site.

### The LPS-Induced Nucleosome Shift between the −2.4 and −2.7 kb Elements Is Transcription Dependent

**Figure 7:**
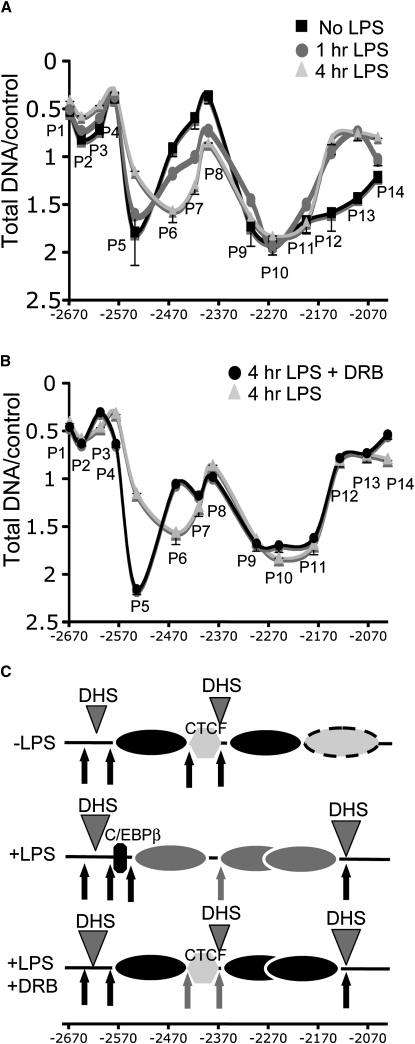
Analysis of Nucleosome Positioning for the −2670/−2050 bp Region Using Real-Time Quantitative PCR Genomic DNA isolated from the mononucleosomal fraction was amplified by real-time PCR as described in methods. Data are representative of at least three independent experiments. (A) Nontreated HD11 cells (black square), 1 hr LPS-treated HD11 (dark gray circle), and 4 hr LPS-treated HD11 cells (light gray triangle). (B) Four hour LPS-treated HD11 cells (light gray triangle); 4 hr LPS-treated HD11 cells with DRB added 15 min after the start of LPS induction (black circle). (C) Nucleosomal organization deduced from (A) and (B). Gray triangles indicate DHSs (Huber et al., 1995). Black and gray arrows indicate MNase accessibility sites (Figure 3A), with changes from black to gray arrows indicating a decrease in hypersensitivity. Superimposed nucleosomes indicate a region in which a nucleosome can occupy several positions in different alleles. P1–P14, primers used to measure the representation of specific DNA fragments after nuclease digestion (Figure S7). Error bars represent ± SD from three measurements. Data are representative of three independent experiments.

We next employed a real-time quantitative PCR assay to examine nucleosome positioning in the presence or absence of transcription. As template, we used genomic DNA purified from the mononucleosome fraction of chromatin from induced and noninduced HD11 cells extensively digested with MNase. Overlapping primers (P1–P14) were designed spanning the −1.9/−2.7 kb region, with each amplicon being 60–65 bp long (Figure S7). This approach allowed us to distinguish between nucleosomal length-protected regions and shorter DNA regions protected by transcription factor complexes associated with nuclease hypersensitive sites. The results were in agreement with our earlier prediction of the predominant nucleosome positions (Figure 3). In unstimulated HD11, maximum DNA amplification was observed with P5 and P9–P11 primers corresponding to the nucleosomal regions between −2540 and −2430 bp, and −2267 and −2200 bp, respectively. In contrast, the CTCF-containing linker region between −2366 and −2430 bp presented a low enrichment with P6, P7, and P8 primers (Figure 7A). Similarly, upstream of the −2597 bp linker MNase site, low DNA enrichment correlated with the presence of the DHS encompassing the −2.7 kb enhancer (P1–P4). Here the amount of nucleosomal material detected further decreased after stimulation, correlating with the full activation of the −2.7 kb enhancer element.

Inducible nucleosome reorganization was also detected in the region flanking the −1.9 kb HRE (P12–P14). While nucleosomes occupied most of the −2310 to −2050 bp region in unstimulated cells, the region from −2100 to −2050 bp was predominantly nucleosome-free in stimulated cells after 1 hr (Figure 7A). This remodeled region corresponds to one of the two nuclease hypersensitive regions seen in Figure 3A that define the inducible −1.9 kb DHS and most likely the boundary of the LINoCR promoter. The CTCF-containing linker region (P6–P8) became progressively more protected in stimulated cells and was predominantly nucleosomal after 4 hr of LPS treatment. We observed a parallel decrease in signal at the −2550 bp position (P5), confirming that LPS induces movement of a nucleosome from a position occupying the C/EBP site to a position occupying the CTCF site.

After DRB treatment, no alteration of nucleosome positioning in noninduced HD11 cells was observed (Figures S8A and S8B). Significantly, DRB treatment 15 min after LPS stimulation blocked relocation of the nucleosome from the C/EBP site to the CTCF site (seen with P5 and P6, Figure 7B). Transcription dependence of nucleosome remodeling at the −2.4 kb element was specific for this region, since DRB treatment had no effect on any other LPS-induced chromatin alterations downstream and upstream these two transcription factor-binding sites. Therefore, DNA amplification with P7 and P8 primers designed in the CTCF-containing linker region significantly increased after LPS and DRB treatment compared to untreated cells, suggesting that full occupancy at −2.7 and −1.9 kb elements restricted the position of the nucleosomes in the vicinity of the −2.4 kb element. These results were independently confirmed by two indirect end-labeling experiments (Figure S8). Taken together, these experiments demonstrate a close link between transcription, nucleosome remodeling at the −2.4 kb element, and CTCF eviction.

## Discussion

### LPS Induction Activates a *cis*-Regulatory Element with Dual Enhancer/Promoter Function

The −1.9 kb promoter/enhancer is an interesting example of an element exerting differential inducible functions in two different tissues (oviduct and macrophages). It was previously shown to be important for the steroid-inducible expression of lysozyme in nonmyeloid cells (Hecht et al., 1988). Here we provide insights into the role of this element in myeloid cells and into the molecular mechanism of its activation. The −1.9 kb element is the only lysozyme 5′ *cis*-regulatory element that is not occupied by transcription factors prior to induction, underlining the importance of the chromatin environment for these proteins to access their DNA-binding sites. It binds AP-1 and C/EBPβ 20 min after LPS induction, and activation is associated with extensive remodeling of the underlying chromatin. Two inducible MNase and DHSs located at −2.1 and −1.9 kb flank the predicted start site at −2.0 kb and most likely represent regions in which nucleosomes have been displaced.

As a promoter, the −1.9 kb element drives the transient expression of a noncoding transcript (LINoCR), which reads through the −2.4 kb element. After LPS induction, transcription factor and RNA Pol II are rapidly and simultaneously recruited to both promoters, and LINoCR expression is detected prior to increased lysozyme mRNA expression. Our CTCF knockdown experiments showing that CTCF/cohesin interferes only with lysozyme mRNA expression explain this timing discrepancy and suggest that LINoCR transcription is a prerequisite for the abrogation of the repressive activity of CTCF. They also demonstrate that this repressive activity is context dependent.

### LPS Stimulation Leads to CTCF Eviction from the −2.4 kb Element

An important result from this study is our finding that CTCF is evicted from its binding site after LPS stimulation. Previous publications have established that CTCF enhancer-blocking functions could be regulated, but without change in CTCF occupancy (El-Kady and Klenova, 2005; Lutz et al., 2003). We also established that Rad21, a protein of the cohesin complex, colocalizes with CTCF at the lysozyme locus as observed at other gene loci (Parelho et al., 2008; Wendt et al., 2008), providing an interesting example of an enhancer-blocking element bisecting a regulatory region and repressing gene expression. Both proteins are evicted from the −2.4 kb element. In yeast, transcription elongation into cohesin-associated sites results in local dissociation of cohesin/chromosome interaction (Bausch et al., 2007). This observation suggests a possible role of LINoCR transcription in the eviction of the CTCF/cohesin complex, and we indeed observed that CTCF and Rad21 eviction from the −2.4 kb element was transcription elongation dependent. LINoCR is highly unstable, and we were unable to knock it down by siRNA, indicating that targeted molecules may be already on their way for degradation. This makes it difficult to completely exclude an effect of LINoCR on CTCF/cohesin eviction in *trans* but supports the idea that transcription itself has a regulatory function. This observation is also consistent with the reported short half-life of other ncRNAs impacting on gene regulation in *cis*, such as the imprinting-regulating antisense transcript Air (Seidl et al., 2006). In addition, we did not observe a delay between LINoCR transcription and histone H3 acetylation/phosphorylation. These data argue in favor of a fast deactivation of the CTCF-associated repressive complex rather than a *trans*-acting effect.

### CTCF Eviction Is Associated with Transcription-Dependent Repositioning of a Nucleosome over the CTCF-Binding Site

Differential CTCF binding has been shown to involve differential methylation at imprinted genes (see, for example, Schoenherr et al., 2003). As the CTCF site at the −2.4 kb element does not contain a CpG, our results argue for an important role of chromatin structure in the eviction of CTCF. Histone H3 phosphorylation has been associated with the eviction of HP1 from heterochromatin during DNA replication (Hirota et al., 2005; Mateescu et al., 2004). However, it is not known whether CTCF, like HP1, is capable of interacting with histone tails. Therefore, elongation-dependent recruitment of the IKKα kinase and phosphorylation at the −2.4 kb element may be associated with the destabilization of a CTCF-associated silencing complex (Lutz et al., 2000). It has indeed been shown that CTCF loses its repressive properties after phosphorylation (El-Kady and Klenova, 2005). However, our analysis of the nucleosome positioning before and after LPS treatment indicates an additional mechanism ensuring permanent CTCF eviction that employs nucleosome repositioning to prevent the reassociation of CTCF with its binding site after LPS stimulation.

Remarkably, DRB treatment only affected CTCF/cohesin eviction and LPS-induced repositioning of the nucleosome over the CTCF-binding site. These observations rule out an unspecific effect of DRB on transcription factor binding, chromatin structure, and LPS signaling. CTCF cannot bind once its target site is covered by a nucleosome (Kanduri et al., 2002). Our data are consistent with the idea that, in the context of the −2.4 kb element, the CTCF/cohesin complex prevents the full activation of the −2.7 kb enhancer and that the passage of the RNA Pol II complex together with histone H3 S10 phosphorylation and a concomitant destabilization of nucleosomes induces a resetting of the chromatin structure over the CTCF-binding site and allows additional C/EBPβ proteins to be recruited to the −2.7 kb enhancer.

In summary, we have identified a mechanism that employs transcription-dependent alterations in chromatin architecture for the inducible regulation of the lysozyme gene (Figure S9). It has been suggested that as much as 98% of the transcriptional input in human genome does not encode for protein (Mattick, 2005). Therefore, the ability of intergenic transcription to alter the regulatory properties of *cis*-elements as exemplified by the lysozyme gene may provide a general mechanism of gene regulation.

## Experimental Procedures

### Cell Culture

The chicken cell lines HD11 (Beug et al., 1979) and HD37 (Graf et al., 1992) were grown in Dulbecco's modified Eagle's medium as previously described (Lefevre et al., 2005). Where indicated, HD11 cells were treated with 5 μg/ml LPS (Sigma) and 200 μM 5,6-dichloro-1-β-D-ribofuranosylbenzimidazole (DRB) (Alexis).

### Reverse Transcriptase-PCR

Total RNA was prepared and standard RT-PCR performed as previously described (Lefevre et al., 2005). Detection of the intergenic transcripts was performed according to a previous publication (Tagoh et al., 2004), using the following biotinylated primers for the cDNA synthesis: U2.4, CTGAATTGCAAAGCAGGAGT; and GAPDH, ATCAGTTTCTATCAGCCTCT. Relative expression was calculated as a ratio of specific transcript to GAPDH (for primer sequences, see Table S1).

### Chromatin Immunoprecipitation Assays and Real-Time PCR Analysis

ChIP was performed exactly as previously described (Lefevre et al., 2003), with 1 μg anti-NFκB (Santa Cruz sc-7151X), anti-histone H3 phospho S10 and acetyl K9 (Abcam ab12181), anti-Fos (Santa Cruz sc-253X), anti-Fli-1 (Santa Cruz sc-356X), anti-CTCF (Upstate Biotechnology), and anti-C/EBPβ antibodies, except for experiments described in Figures 2D, 5A, and 6. For these two experiments, samples were diluted with IP buffer containing 0.3% SDS to obtain a final solution of 10^7^ cells/ml in 0.2% SDS. Chromatin was sonicated 2 × 15 min using a Bioruptor 200 (Diagenode). Prior to sonication, Dynabeads protein A (Invitrogen) were washed twice with IP buffer containing 0.2% SDS. Beads (10 μl) were added to 90 μl IP buffer with 0.2% SDS and 1 μg anti-RNA Pol II (Santa-Cruz sc-900X), anti-AcK9 (Abcam ab444-1), anti-CTCF (Upstate Biotechnology 07-729), anti-Rad21 (Abcam ab992), or IgG control (Upstate 12-370) in a PCR plate and incubated on a rotator for 2 hr at 4°C. Sonicated chromatin (100 μl) was transferred to the PCR plate containing the antibody-bead complexes and rotated again for 2 hr at 4°C. After immunoprecipitation, beads were washed and eluted as previously described. The rest of the procedure, including primers used for Real-time PCR quantification, was as described in Lefevre et al. (2003).

### Nucleosome Mapping by Indirect End Labeling

DNase I treatment of cells and naked DNA was performed as previously described (Lefevre et al., 2005). MNase digestions of HD11 and indirect end labeling were performed using isolated nuclei as described previously (Johnson et al., 2004). With 10 μg of each, different DNA preparations digested with 20U SphI (New England Biolabs) for 3 hr at 37°C and stopped with 5× loading dye 20% Ficoll (Sigma), 1% SDS (Sigma), and 0.05% bromophenolblue (Sigma). The probe abutting the Sph I site (−3165 to −2865 bp) was prepared by PCR using a plasmid containing the full sequence of the lysozyme locus as a template with the following primers: fwd, TACTTAGGAGGGTGTGTGTG, and rev, GCACCTTGAAGATTTGTT. The probe was gel purified using a QIAquick Gel Extraction Kit (QIAGEN).

### Stealth RNAi Transfection

Transfection was performed according to the manufacturer's recommendations (Invitrogen). Briefly, HD11 cells were trypsinized and resuspended at the concentration of 2 × 10^5^ cells/400 μl of normal medium. Lipofectamine 2000 (2.5 μl) was diluted in 50 μl Opti-MEM reduced serum medium without serum (Invitrogen). After 5 min incubation, this solution was added to 60 pmol of Stealth RNAi oligomer previously diluted in 50 μl of the same medium. After 20 min incubation, oligomer-Lipofectamine 2000 complexes were added to each well of a 24-well plate containing 400 μl of cells and medium. LPS stimulation was carried out 24 hr later. A cocktail of 3 × 20 pmol of Stealth RNAi oligomers was used to target CTCF (see Table S1).

### Transient Transfection

DNA fragments carrying the lysozyme promoter (−376 to +17 bp) and the −1.9 kb element (−2132 to −1877 bp) were cloned in both orientations into the luciferase vector pXPG (Bert et al., 2000). Transfection was performed using jetPEI (polyplus/Qbio gene) and the dual reporter luciferase assay system (Promega). HD11 cells were seeded at 1.5 × 10^5^ cells/well for 24-well plates in 500 μl of culture medium. Twenty-four hours prior to transfection, 0.25 μg/well test plasmid and 150 pg/well Renilla luciferase plasmid (pRL-CMV) were mixed with 0.5 μl/well JetPEI into 100 μl of 150 mM NaCl. After 30 min, the solution was added to the cells and incubated for 2 hr. Medium (2 ml) was added and cells were incubated for an additional 11 hr before stimulation with 5 μg/ml LPS for 7.5 hr before performing standard luciferase assays.

### Nucleosome Mapping Using Real-Time Quantitative PCR

Genomic DNA purified from the mononucleosome fraction was prepared as previously published (Lefevre et al., 2005). Real-time PCR was performed by using primer pairs listed in Figure S7, and the DNA concentration of each amplicon was determined by comparison to a serial dilution of genomic DNA. Data are expressed using the following formula, [concentration]_amplicon_/average([concentration]_contolA_ and [concentration]_contolB_), with control primers A and B designed within the Apovldl2 promoter and 10 kb upstream the lysozyme TSS, respectively (Lefevre et al., 2005).

## Supplemental Data

Document S1. Nine Figures and One Table
