# Supplementary material for: The LPS-Induced Transcriptional Upregulation of the Chicken Lysozyme Locus Involves CTCF Eviction and Noncoding RNA Transcription
Source: Mol Cell. 2008 Oct 10;32(1-24):129–39. doi: 10.1016/j.molcel.2008.07.023 (PMC2581490; doi:10.1016/j.molcel.2008.07.023)
Supplement: Document S1. Nine Figures and One Table [file mmc1.pdf]

Molecular Cell, Volume *32*

## Supplemental Data

# The LPS-Induced Transcriptional Upregulation of the Chicken Lysozyme Locus Involves CTCF Eviction and Noncoding RNA Transcription

**Pascal Lefevre, James Witham, Claire E. Lacroix,**
**Peter N. Cockerill, and Constanze Bonifer**

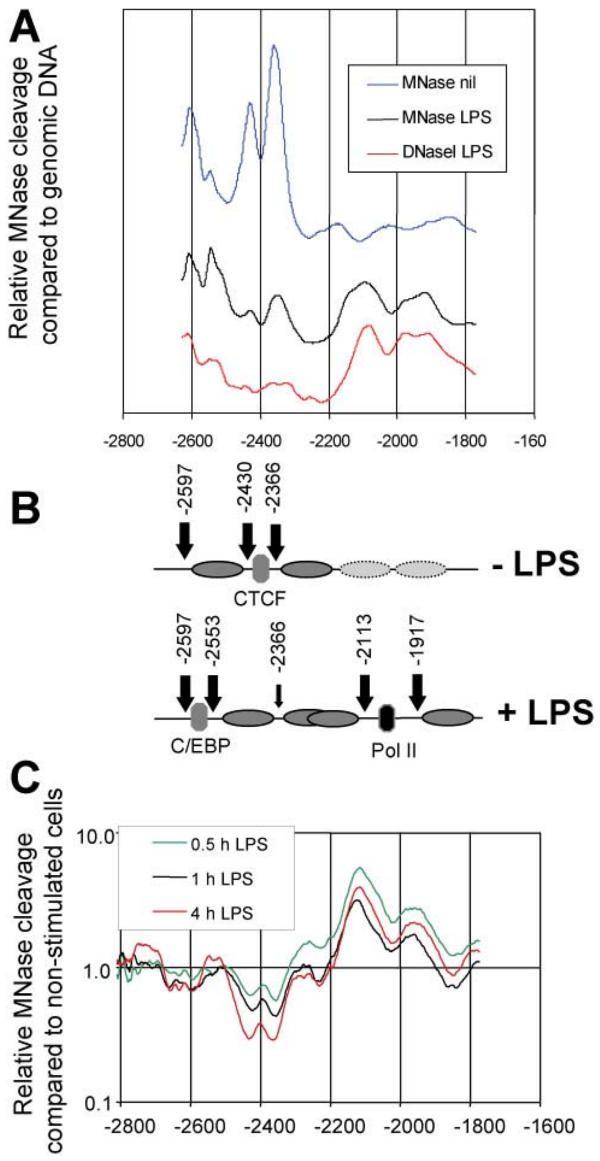

**Figure S1: Densitometric analyses of relative rates of MNase cleavage within chromatin at the -2.7/-1.9 kb region.** Densitometric traces were generated from the analyses depicted in figure 3A, and the data was digitally transformed so that it could be plotted on a linear scale with respect to the position of each data point within the sequence. This was achieved with the aid of a standard curve derived from the migration rates of a molecular weight marker. (A) The relative rate of MNase cleavage at each point of the curves was calculated by dividing the values obtained for MNase digestions of cells by the values obtained by digesting genomic DNA, as in previous study (Bert et al. 2007). Profiles of the relative MNase cleavage for cells treated for 0 and 4 hrs with LPS are shown as the top 2 traces, and underneath is the trace obtained for the actual level of DNase I cleavage obtained from cells stimulated for 6 h with LPS. (B) Locations of nucleosomes (ovals) and hypersensitive sites (large arrows) derived from the data in panel A. The small arrow indicates a linker region that defines the boundary of a relocated nucleosome. The less distinct ovals indicate the preferred positions of nucleosomes, which are not strictly fixed. (C) Relative changes in the rate of MNase cleavage induced by LPS at each position were calculated by dividing values obtained from stimulated cells by the values obtained from non-stimulated cells. A value of 1 represents no difference in the signals obtained between the samples.

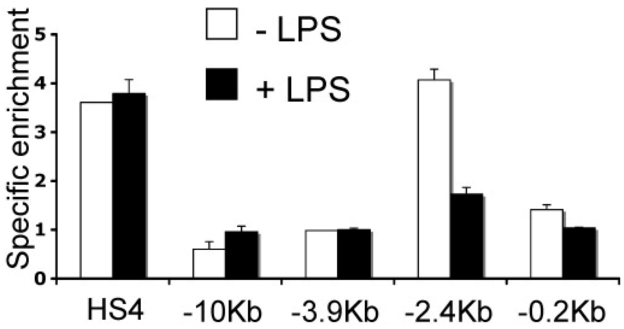


**Figure S2: LPS-induced CTCF removal is chicken lysozyme specific.** ChIP assay with an anti-CTCF antibody able to react with chicken CTCF. HD11 cells were left untreated or treated with LPS for 4 hours. Data are analyzed by real-time PCR with primer pairs named according to their distance from the lysozyme gene transcription start site or specific for the chicken β-globin HS4 (supplementary table). Error bars represent ±SD from three measurements.

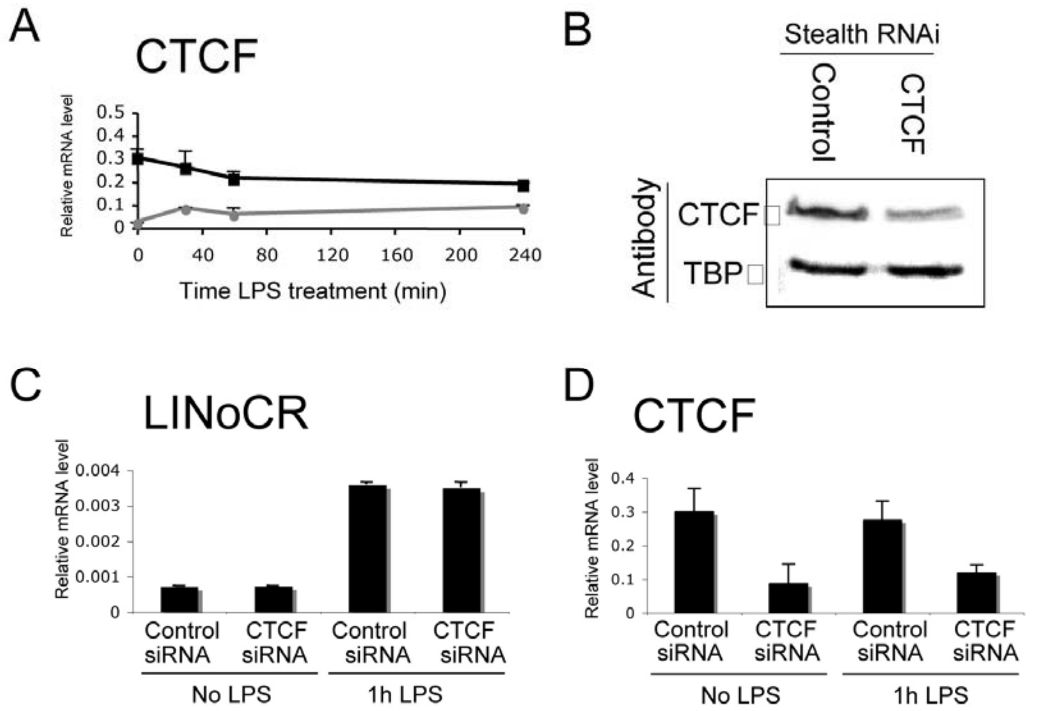


**Figure S3: CTCF knockdown with stealth RNAi (Invitrogen).** (A) Time course of CTCF gene expression following CTCF knockdown in HD11 cells (grey circles) compared to the control (black squares). (B) Western Blot of CTCF protein level following CTCF knockdown in HD11 cells. This experiment was performed as previously described (Tagoh et al., 2006) with anti-CTCF (abcam ab-10571) and anti-TBP (Diagenode). (C) Effect of CTCF knockdown on LINoCR expression without or after 1h LPS treatment. (D) Effect of CTCF knockdown on CTCF expression without or after 1h LPS treatment. Data are expressed relative to GAPDH and error bars represent ±SD from three independent experiments (For primer sequences see supplementary table).

**A**

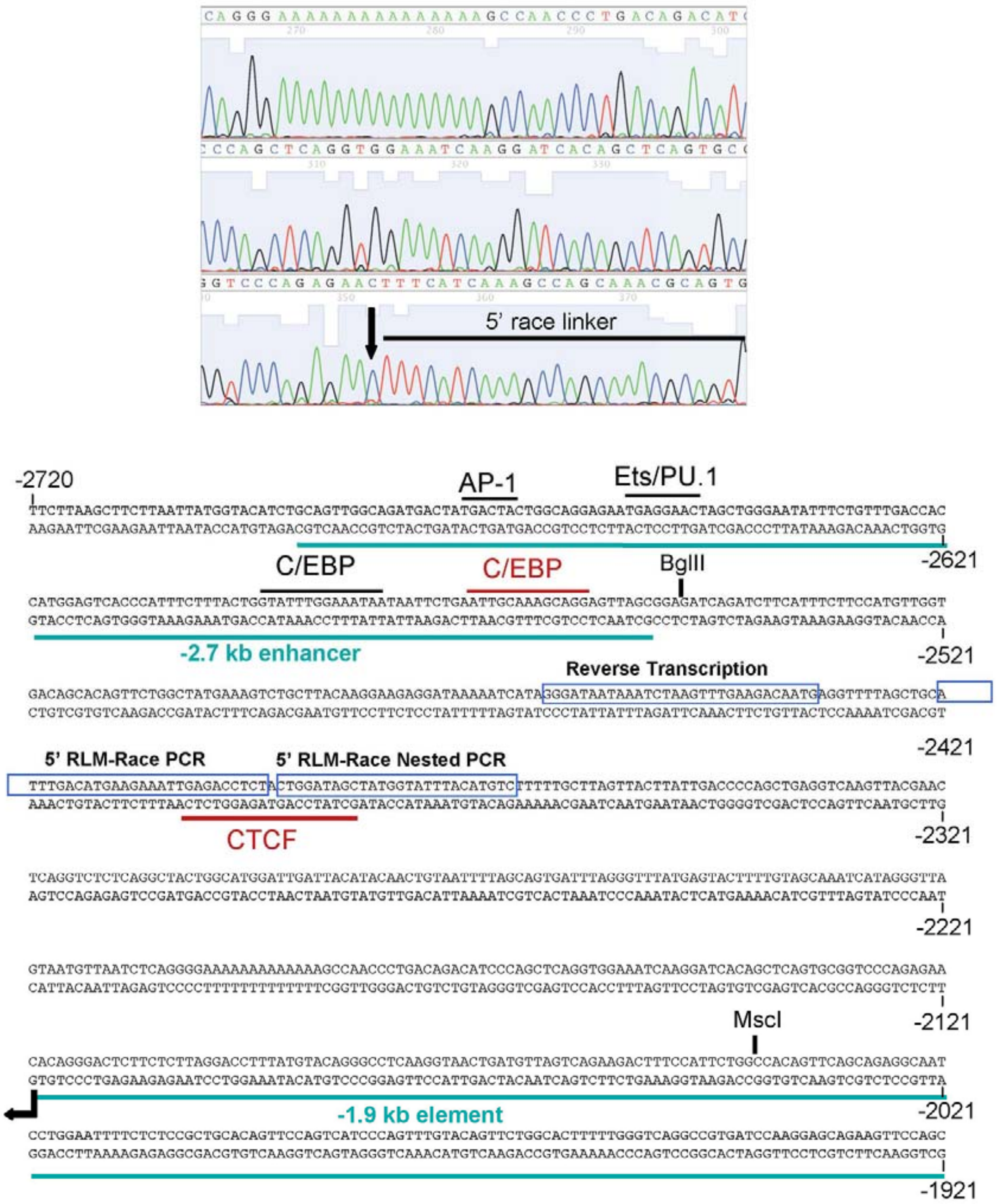
5' race linker
-2720
AP-1
Ets/PU.1
-2621
C/EBP
C/EBP
BglII
-2.7 kb enhancer
-2521
Reverse Transcription
-2421
5' RLM-Race PCR
5' RLM-Race Nested PCR
CTCF
-2321
-2221
MscI
-2121
-1.9 kb element
-2021
-1921

**B**

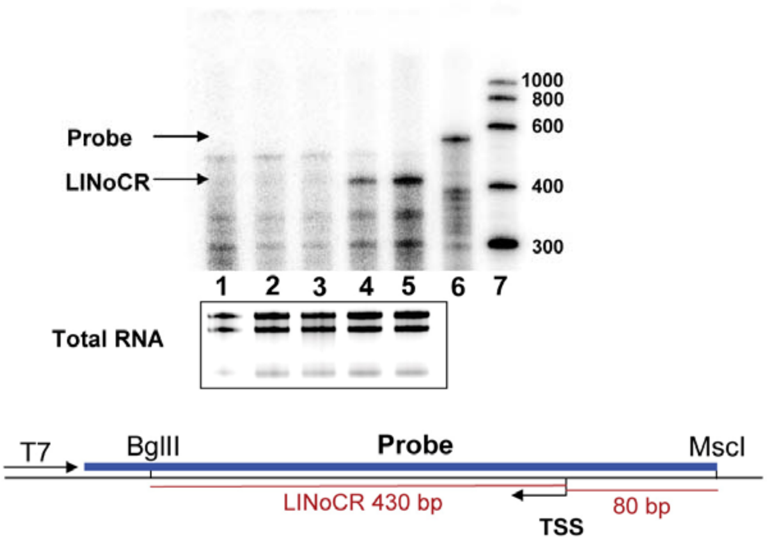


**Figure S4: Localization of the LINoCR transcription start site.** (A) 5'RACE analysis was performed using the FirstChoice® RLM-RACE kit (ambion) according to manufacturer recommendations. The final PCR product was purified and cloned using a TA cloning kit (invitrogen). 20 clones were sequenced with M13 forward and reverse primer and revealed a unique transcription start site (black arrow). Sequence of the -2720 bp/-1921 bp region containing the CTCF and C/EBP binding sites (in red) and the transcription start site (black arrow). The blue boxes represent primers designed for the 5' race analysis. The sequences highlighted in green represents the position of the -1.9 kb and -2.7 kb elements. (B) Identification of LINoCR using RNase protection assay. This experiment was performed using a RPA $II^{TM}$ kit (Ambion) according to manufacturer recommendations. A fragment spanning from -2.54 kb to -1.56 kb of the lysozyme locus was cloned into pBluescript KS(+) (Stratagene) cut with BamHI and PstI. The probe for the RNase protection assay was generated by in vitro transcription using T7 RNA polymerase and $[^{\alpha 32}P]$ UTP using the same plasmid linearized with MscI as template (as detailed in blue at the bottom part of the figure). 100 μg of total RNA isolated from the erythroblast cell line HD37 (lane 1), HD11 cells (lane 2 and 3) and HD11 cells treated with LPS for 1hr (lane 4 and 5) was incubated with the probe overnight at 42°C and then treated with RNase. The non-treated control showing the size of the full-length probe is shown in lane 6 as

well as a labeled RNA marker (sigma R6895) (lane 7). Equal input was checked on an agarose gel stained with ethidium bromide (lower panel). Black arrows indicate the position of the bands corresponding to the probe and LINoCR RNA fragment. The expected size of the LINoCR fragment is 430 bp as depicted in the bottom part of the figure.

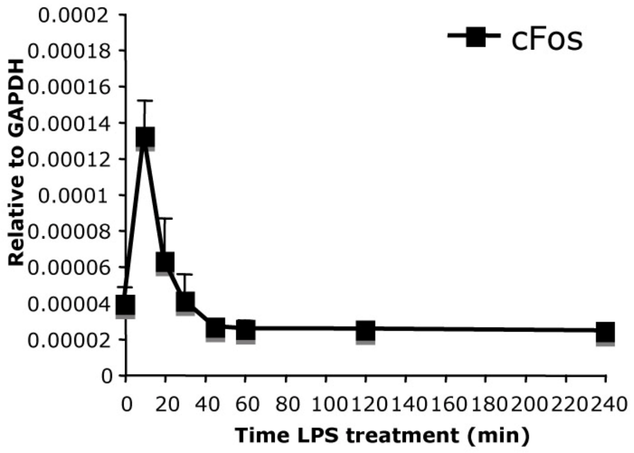


**Figure S5:** Time course of cFos expression following LPS stimulation of HD11 cells. Results are expressed relative to GAPDH expression and error bars represent ±SD from three independent experiments (For primer sequences see supplementary table).

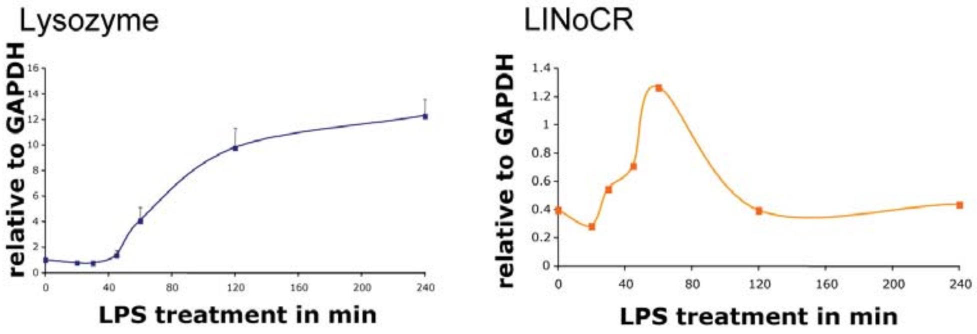


**Figure S6: The lysozyme and LINoCR expression profile is conserved in transgenic primary mouse macrophages.** Chicken lysozyme and LINoCR expression analysis in primary macrophages from transgenic mice harboring a single copy of the complete lysozyme locus depicted in figure 1. Cells were left untreated or treated with LPS for up to 4 h. Results are expressed relative to mouse *gapdh* expression (supplementary table) and error bars represent ±SD from three independent experiments.

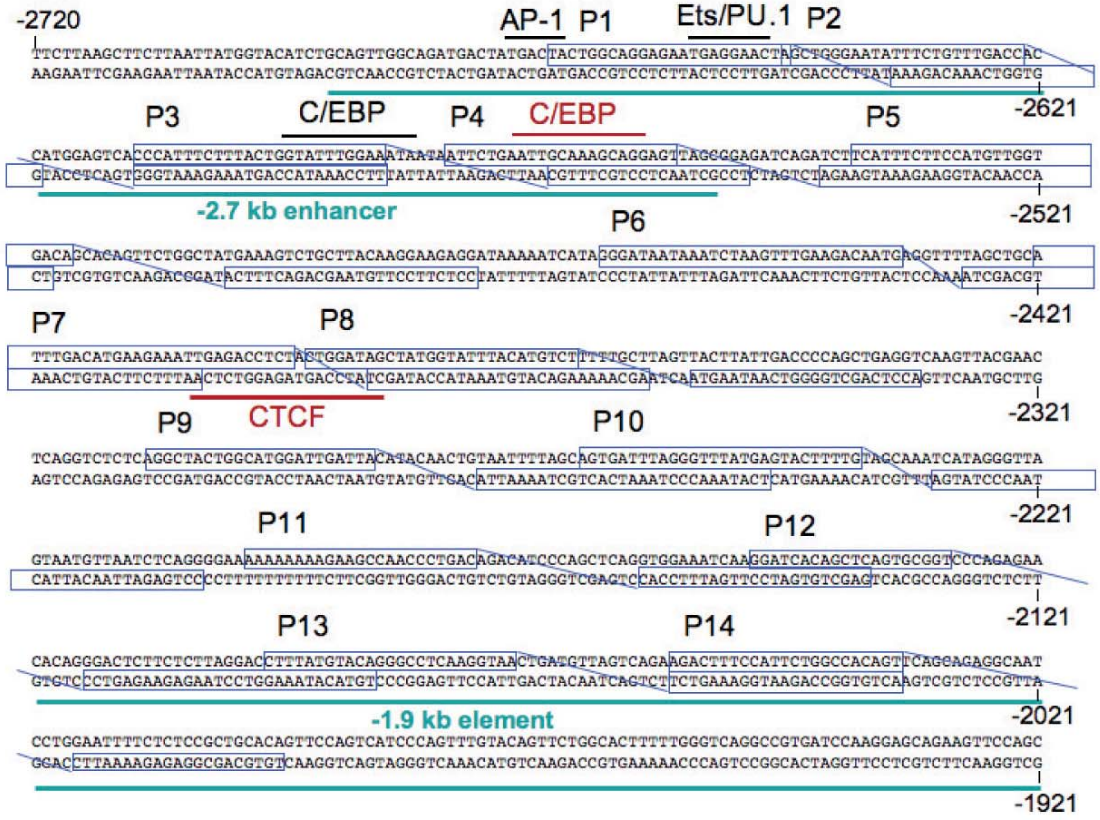


**Figure S7: Primer designed for nucleosome positioning analysis by real-time quantitative PCR.** The displayed sequence is part of the 5’ regulatory region of the lysozyme locus between 2720 bp and 1921 bp upstream the transcription start site. The sequences for primer pairs P1 to P14 are enclosed by blue rectangles. CTCF and C/EBP consensus sequences are highlighted in red, other characterized sites at the -2.7 kb enhancer are indicated in black, and the -2.7 kb and -1.9 kb elements are underlined in green.

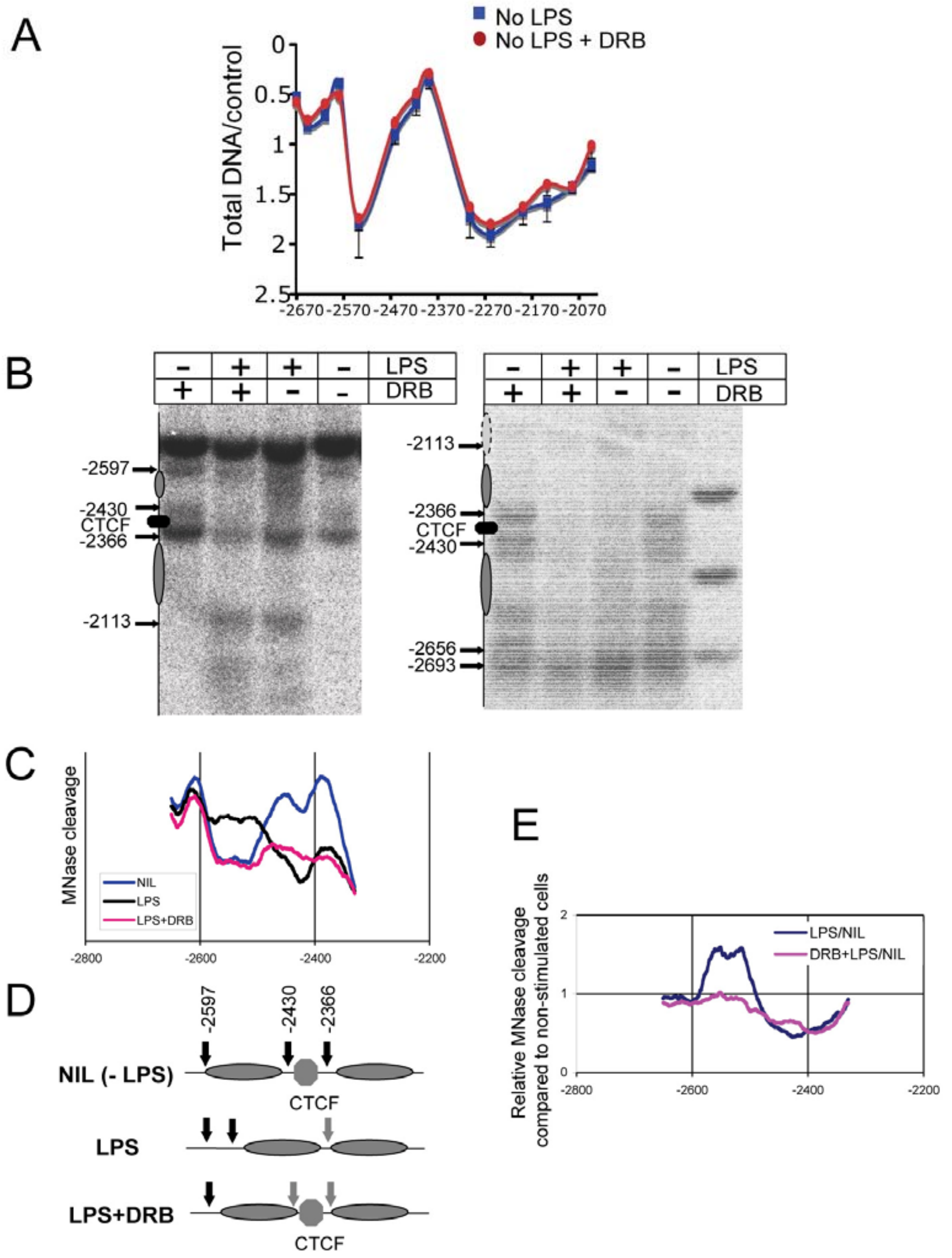


**Figure S8: DRB blocks nucleosome repositioning over the CTCF binding site.** (A) Genomic DNA isolated from the mononucleosomal fraction was amplified by real-time PCR as described in methods. Non-induced HD11 cells with (red circle) or without (dark blue square) were treated with DRB for one hour. Error bars represent ±SD from three independent experiments. (B) MNase digestion analysis of a region 3 kb upstream the transcription start site. Genomic DNA isolated from MNase treated chromatin was analyzed as described in methods. HD11 cells were treated with LPS

for 0 and 240 min with or without DRB as indicated at the top of the figure. The nucleosomal organization for non-induced HD11 cells is depicted on the left part of the figure. Left panel: 3’ to5’ direction, right panel: 5’ to 3’ direction. The black arrows indicate the position of MNase hypersensitive sites described in figure 3. (C) Densitometric analyses of relative rates of MNase cleavage within chromatin at the -2.7/-1.9 region generated from the analyses depicted in figure S8B right panel. The data was digitally transformed so that it could be plotted on a linear scale with respect to the position of each data point within the sequence. This was achieved with the aid of a standard curve derived from the migration rates of a molecular weight marker (right panel). Profiles of the relative MNase cleavage for cells treated for 0 (blue) and 4 hrs with LPS (black) or with 4 hrs LPS + DRB. (D) Locations of nucleosomes (ovals) and hypersensitive sites (arrows) derived from the data in panel C. Changes from black to grey arrows indicate a decrease in hypersensitivity compare to unstimulated cells. (E) Relative changes in the rate of MNase cleavage induced by LPS (blue) or LPS + DRB (red) at each position were calculated by dividing values obtained from stimulated cells by the values obtained from non-stimulated cells. A value of 1 represents no difference in the signals obtained between the samples.

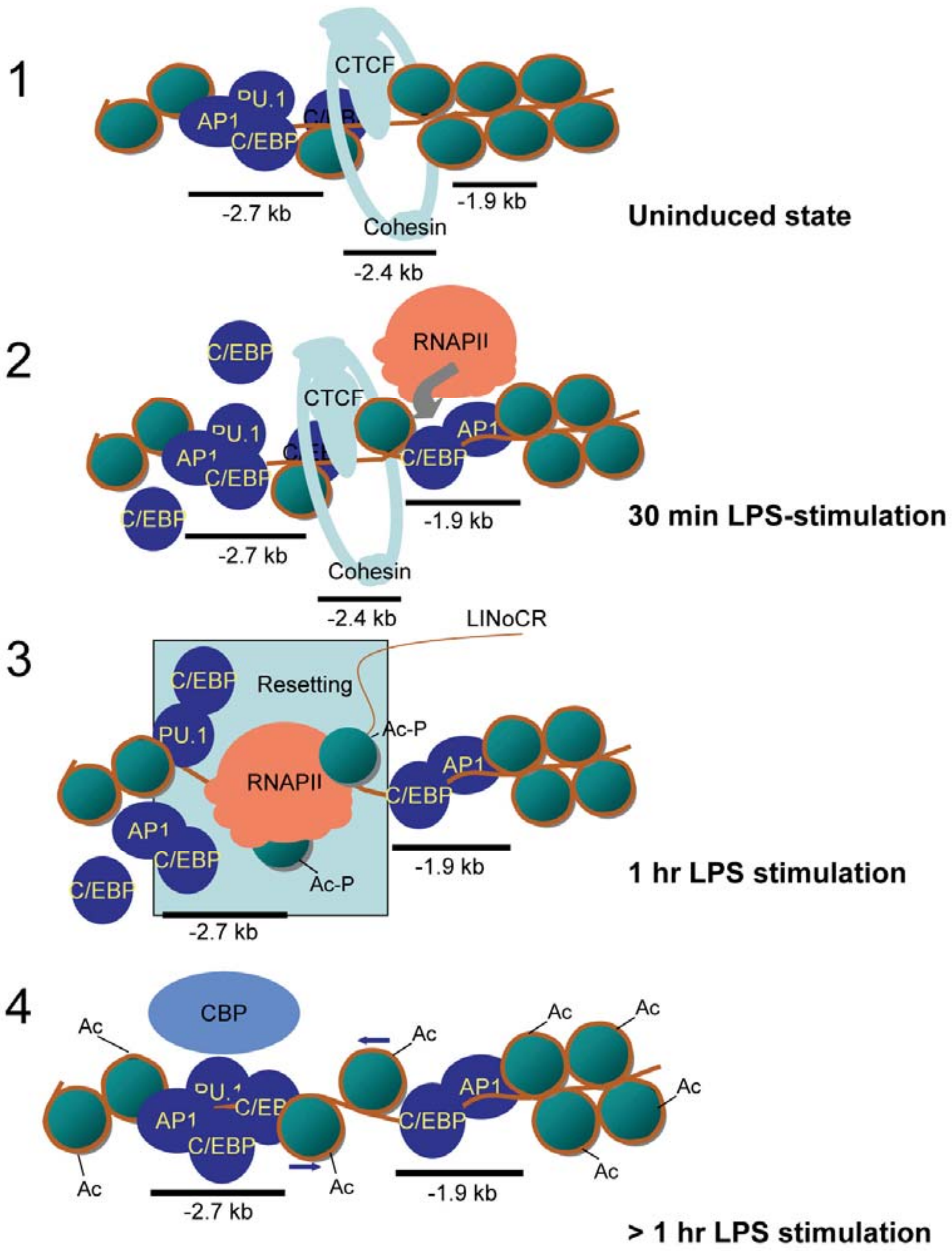


**Figure S9: Model of LPS induced chromatin reorganisation of the -1.9 kb/-2.7 kb region.** (1) In monocytes, the CTCF/cohesin complex occupies a large nucleosome linker region at the lysozyme silencer. The upstream enhancer (-2.7 kb) is occupied by the indicated transcription factors but does not recruit the co-activator CBP (Lefevre et al., 2003). (2) Short LPS treatment (30 min) induces binding of C/EBPb and AP1 to a newly activated cis-regulatory element (-1.9 kb) with dual enhancer-promoter function and RNA Pol II is recruited. (3) The LPS Inducible Non-coding

RNA (LINoCR) is transcribed and the nucleosomal organisation over the -2.4 kb/-2.7 kb region is altered. In addition, nucleosomes are transiently phospho-acetylated exclusively over the transcribed region. (4) This leads to the re-positioning of a nucleosome over the CTCF binding site. The CTCF/cohesin complex cannot bind anymore and CBP is recruited at the -2.7 kb enhancer resulting in a further increase in acetylation. Lysozyme mRNA transcription is upregulated.

# Table S1: Primers list

| Function | Location | Sequence |
|---|---|---|
| Stealth™ RNAi oligomers | Chicken CTCF | GCCAGUGUGGAGGUUAGCAAAUUGA<br>CAGUGUGGAGGUUAGCAAAUUGAAA<br>CCAGCAGGGAUACUUACAAACUGAA |
| Stealth™ RNAi oligomers | Control | GCCUGUAGGUGGGAUAACUAAGUGA |
| Expression analysis | Chicken lysozyme | Forward GAAGCGTCACGGACTTGATAACTA<br>Reverse CCCATCGGTGTTACGGTTTG |
| Expression analysis | Chicken cFos | Forward GGGCTCCCTGAACTCTGCTT<br>Reverse CCAACGCACCCTACGCTTAG |
| Expression analysis | Chicken CTCF | Forward TGCCACCCAAACCAAACAAT<br>Reverse ACTATAATGTTTTCGATTTCACCAGTGT |
| Expression analysis | Chicken GAPDH | Forward GCCAGTTCTGTTCCCTTCTGTCT<br>Reverse CTCTCCCACCTCCCCTAGGT |
| Expression analysis | Mouse GAPDH | Forward AAATCCGTTTCACACCGACCTT<br>Reverse ACAGCCGCATCTTCTTGTGC |
| Biotinylated primer for RT | Mouse GAPDH | GCAGCCCTGGTGACCAGGCGCCCAATACGG |
| Non-coding RNA detection | A | Forward TCAAGTTACGAACTCAGGTCTCTCA<br>Reverse AAACCCTAAATCACTGCTAAAATTACAGT |
| Non-coding RNA detection | B | Forward CCACAGTTCAGCAGAGGCAAT<br>Reverse CCCAAAAAGTGCCAGAACTGTAC |
| ChIP | Chicken HS4 β-globin | Forward GGAGAGGCAGATCTTGGGATAA<br>Reverse TGAACGCTGTGACTTGGAGTGT |
